# Supplementary material for: Pilot Study of Aerosolised Plus Intravenous Vancomycin in Mechanically Ventilated Patients with Methicillin-Resistant Staphylococcus Aureus Pneumonia
Source: J Clin Med. 2020 Feb 9;9(2):476. doi: 10.3390/jcm9020476 (PMC7073531; doi:10.3390/jcm9020476)
Supplement: Supplementary file 1 [file jcm-09-00476-s001.pdf]

Table S1. Inclusion and exclusion criteria.

Inclusion	Exclusion
Age > 19 years	Non-bacterial pneumonia
Mechanical ventilation (≥ 48 hours)	Underlying respiratory condition[c]
Treating intravenous vancomycin	Bronchiectasis
Nosocomial MRSA pneumonia[a]	Previous pulmonary tuberculosis
Healthcare-associated pneumonia	Chronic obstructive pulmonary disease
Hospital-acquired pneumonia	Asthma
Ventilator-associated pneumonia	Pleural effusion required drainage
Community acquired MRSA pneumonia[b]	Active primary or metastatic lung cancer
	Severe congestive heart failure[d]
	Decompensated liver cirrhosis
	Treating intravenous vancomycin rather than pneumonia
	History of adverse effects to inhaled salbutamol
	Hypersensitivity to vancomycin
	Microbiologic evidence of *Enterococcus*
	Pregnancy or breast feeding
	Immunocompromised[e]
	Aggressive medical condition[f]
	Severe acute respiratory distress syndrome[g]
	Vancomycin MIC ≥ 2 mcg/mL
	Other inhaled antibiotics within 48 hours
	Unable to adhere to ventilator protocol
	Unable to perform arterial catheterisation

MRSA, methicillin-resistant *Staphylococcus aureus*; MIC, minimum inhibitory concentration. [a]Definition and classification of nosocomial pneumonia were based on 2005 ATS/IDSA guidelines with MRSA in bronchoalveolar lavage (for the most infiltrative lesion on chest X-ray) fluid $>10^4$ CFU/mL; 'healthcare-associated' means those with pneumonia who had any of the following risk factors: residence in a nursing home or extended care facility; 'hospital-acquired' means pneumonia not incubating at the time of hospital admission and occurring ≥48 hours after admission; and 'ventilator-associated' means that pneumonia occurring ≥48 hours after endotracheal intubation [b]Similar to nosocomial cases, only admitted via emergency room from community (not including healthcare-associated facilities) [c]Confirmation required by computed tomography, spirometry, and/or provocation test; [d]Left ventricular ejection fraction <30% in echocardiography, [e]Systemic corticosteroid (≥ 1 mg/kg) over 3 months, organ transplantation recipient, acquired immune deficiency syndrome, [f]Expected poor prognosis (e.g., Acute Physiologic And Chronic Health Evaluation (APAHCE) II score>35), [g]PaO_2/FiO_2<100 mmHg.

Table S2. Protocol for aerosolised vancomycin treatment.

Drug	Vancomycin HCl 500 mg mixed with isotonic saline 10 mL
Nebulising device	Vibrating mesh plate
Frequency	250 mg/5 mL nebulising for 30 minutes every 12 hours
Duration	5 days
Premedication	Salbutamol 2.5 mg inhalation for 15 minutes
Mechanical ventilator setting[a]	Constant inspiratory flow<br>Tidal volume 6-8 mL/kg (predicted body weight)<br>I:E ratio 1:1.5-1:2.5<br>Plateau pressure < 30 cm H_2O<br>Remove any humidification system and optimise alveolar recruitment<br>Proper sedation

[a]Humidification decreases the amount of drug delivered to the patient secondary to water in the air causing the droplets to clump together and more readily attach to the wall of the tube.

Table S3. Comparison of major outcomes between matching adjusted enrolled and excluded group[a].

Outcomes	Enrolled (n=17)	Excluded (n=17)	P
Age, median (range)	75.5 (47-87)	78 (64-87)	0.547
Sex, male	11 (64.7)	15 (88.2)	0.112
Acute physiology and chronic health evaluation II	23.1 ± 6.9	23.3 ± 6.9	0.921
Clinical response			0.167
Cure	4 (23.5)	0 (5.9)	
Improvement	7 (41.2)	8 (47.1)	
Failure	3 (17.6)	6 (35.3)	
Indeterminate	3 (17.6)	3 (17.6)	
Microbiological response			0.500
Eradicated	12 (70.6)	11 (64.7)	
Persistent	5 (29.4)	6 (35.3)	
Relapse	0 (0.0)	0 (0.0)	
Superinfection	0 (0.0)	0 (0.0)	
Indeterminate	0 (0.0)	0 (0.0)	
Success of treatment	11 (64.7)	8 (47.1)	0.245
Duration of intravenous vancomycin	15.5 ± 5.9	13.1 ± 5.1	0.224
Morality			
In-intensive care unit	5 (29.4)	6 (35.3)	0.500
In-hospital	6 (35.3)	7 (41.2)	0.500

Data are presented as n (%) or mean ± standard deviation, [a]Acute physiology and chronic health evaluation II score and age were matched.

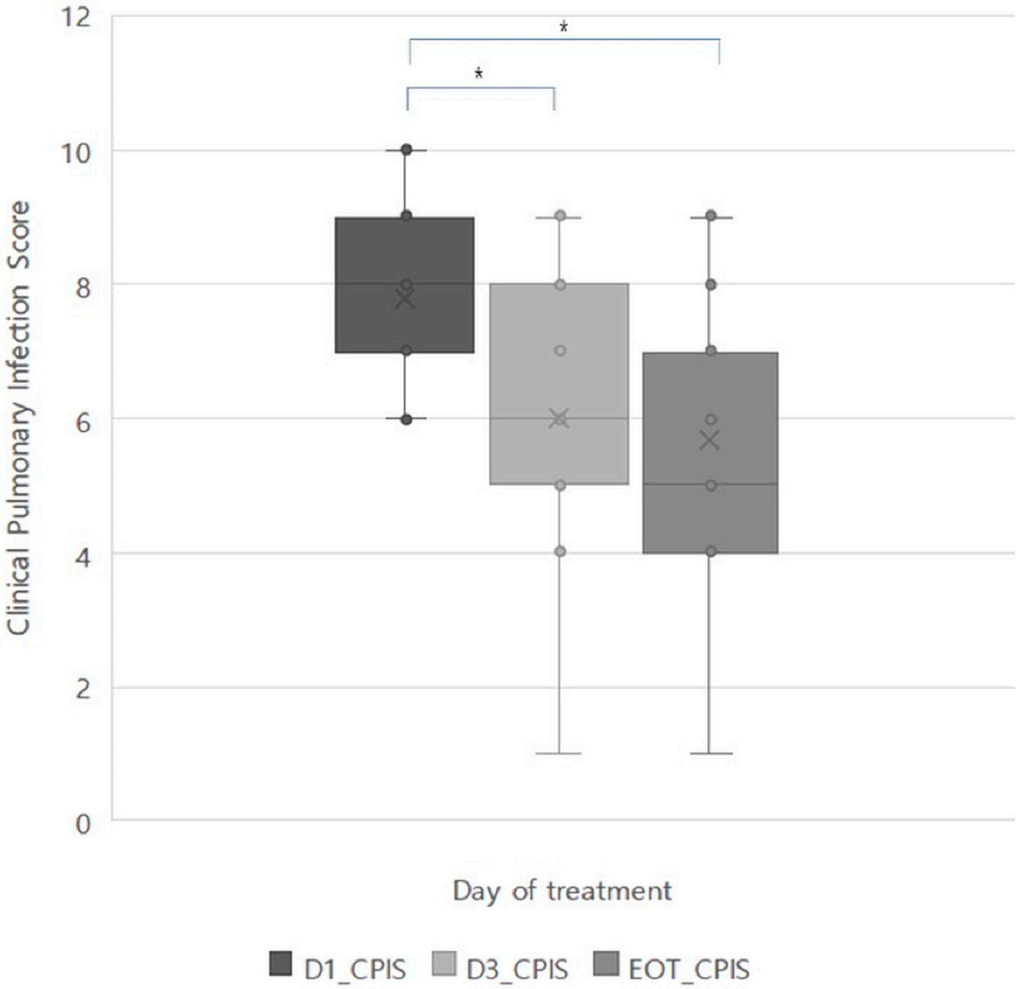

Figure S1. Change in clinical pulmonary infection score.
